# Supplementary material for: Hot spot prediction in protein-protein interactions by an ensemble system
Source: BMC Syst Biol. 2018 Dec 31;12(Suppl 9):132. doi: 10.1186/s12918-018-0665-8 (PMC6311905; doi:10.1186/s12918-018-0665-8)
Supplement: Supplementary file 2 — All relevant MATLAB code. A simple Matlab implement of our predictor. (ZIP 19,742 kb) [file 12918_2018_665_MOESM2_ESM.zip › Additional file 2/Read ASA/ASA website/dbMPIKT/4OFYA.html]

 NetSurfP - Protein Surface Accessibility and Secondary Structure Predictions

  

|  |  |  |
| --- | --- | --- |
|  |  | NetSurfP - Protein Surface Accessibility and Secondary Structure Predictions  Technical University of Denmark |

  


---

```
# For publication of results, please cite:
# A generic method for assignment of reliability scores applied to solvent accessibility predictions.
# Bent Petersen, Thomas Nordahl Petersen, Pernille Andersen, Morten Nielsen and Claus Lundegaard
# BMC Structural Biology 2009, 9:51 doi:10.1186/1472-6807-9-51
#
# Column 1: Class assignment - B for buried or E for Exposed - Threshold: 25% exposure, but not based on RSA
# Column 2: Amino acid
# Column 3: Sequence name
# Column 4: Amino acid number
# Column 5: Relative Surface Accessibility - RSA
# Column 6: Absolute Surface Accessibility
# Column 7: Z-fit score for RSA prediction
# Column 8: Probability for Alpha-Helix
# Column 9: Probability for Beta-strand
# Column 10: Probability for Coil
E A  4OFYA                  1    0.730  80.479  -2.251   0.003   0.003   0.994
E D  4OFYA                  2    0.732 105.539  -0.713   0.018   0.047   0.935
E P  4OFYA                  3    0.501  71.064  -1.506   0.018   0.088   0.893
E Q  4OFYA                  4    0.592 105.695  -0.520   0.020   0.205   0.775
E Q  4OFYA                  5    0.627 111.964   0.116   0.021   0.279   0.699
B L  4OFYA                  6    0.248  45.464   0.608   0.020   0.205   0.775
E Q  4OFYA                  7    0.505  90.211   0.305   0.018   0.088   0.893
B Q  4OFYA                  8    0.195  34.863  -0.252   0.022   0.359   0.619
E R  4OFYA                  9    0.515 117.935   1.208   0.003   0.718   0.279
B I  4OFYA                 10    0.095  17.556   0.073   0.001   0.900   0.099
E V  4OFYA                 11    0.407  62.633   0.439   0.002   0.816   0.182
E E  4OFYA                 12    0.400  69.915   0.388   0.003   0.718   0.279
E A  4OFYA                 13    0.501  55.210   0.720   0.005   0.262   0.733
E P  4OFYA                 14    0.273  38.682  -1.082   0.004   0.085   0.910
E K  4OFYA                 15    0.599 123.297   0.475   0.004   0.197   0.799
E D  4OFYA                 16    0.557  80.278   0.111   0.004   0.616   0.381
B T  4OFYA                 17    0.137  19.030   1.227   0.001   0.900   0.099
E L  4OFYA                 18    0.447  81.864   1.302   0.001   0.959   0.040
B A  4OFYA                 19    0.064   6.998  -0.188   0.001   0.959   0.040
E A  4OFYA                 20    0.462  50.956   1.100   0.002   0.816   0.182
E V  4OFYA                 21    0.441  67.843   0.988   0.005   0.262   0.733
E G  4OFYA                 22    0.659  51.848  -0.785   0.005   0.015   0.979
E E  4OFYA                 23    0.443  77.479   1.237   0.004   0.085   0.910
E T  4OFYA                 24    0.517  71.722   1.336   0.003   0.718   0.279
B A  4OFYA                 25    0.035   3.846   0.213   0.001   0.959   0.040
E I  4OFYA                 26    0.364  67.340   1.916   0.001   0.959   0.040
B L  4OFYA                 27    0.037   6.701   0.569   0.001   0.959   0.040
E T  4OFYA                 28    0.370  51.333   1.786   0.001   0.959   0.040
B C  4OFYA                 29    0.016   2.317   1.118   0.001   0.900   0.099
E R  4OFYA                 30    0.396  90.798   1.783   0.001   0.959   0.040
B V  4OFYA                 31    0.052   7.977  -0.957   0.001   0.959   0.040
E E  4OFYA                 32    0.519  90.582   0.605   0.004   0.616   0.381
E H  4OFYA                 33    0.691 125.620  -1.468   0.004   0.085   0.910
E Q  4OFYA                 34    0.535  95.622   0.028   0.005   0.045   0.951
E Q  4OFYA                 35    0.548  97.926  -1.375   0.005   0.045   0.951
E G  4OFYA                 36    0.403  31.677  -0.033   0.005   0.336   0.660
E P  4OFYA                 37    0.304  43.152   0.743   0.003   0.718   0.279
B V  4OFYA                 38    0.031   4.765   0.472   0.001   0.900   0.099
B Q  4OFYA                 39    0.211  37.595   1.584   0.001   0.959   0.040
B W  4OFYA                 40    0.027   6.590   0.475   0.006   0.962   0.032
B M  4OFYA                 41    0.173  34.637   0.957   0.001   0.959   0.040
B K  4OFYA                 42    0.207  42.600   0.844   0.002   0.816   0.182
E D  4OFYA                 43    0.539  77.670  -0.131   0.005   0.045   0.951
E D  4OFYA                 44    0.695 100.207  -2.392   0.005   0.015   0.979
E F  4OFYA                 45    0.472  94.670  -0.141   0.003   0.718   0.279
E G  4OFYA                 46    0.360  28.293  -0.400   0.002   0.816   0.182
B L  4OFYA                 47    0.126  23.107  -0.236   0.021   0.756   0.223
E G  4OFYA                 48    0.310  24.381  -0.319   0.023   0.655   0.322
E T  4OFYA                 49    0.340  47.130  -1.578   0.004   0.420   0.576
E D  4OFYA                 50    0.321  46.184  -1.583   0.020   0.205   0.775
E R  4OFYA                 51    0.567 129.774  -2.182   0.019   0.141   0.840
E D  4OFYA                 52    0.504  72.583  -1.490   0.022   0.359   0.619
E K  4OFYA                 53    0.314  64.549  -0.368   0.003   0.718   0.279
E P  4OFYA                 54    0.433  61.400  -0.239   0.021   0.756   0.223
B L  4OFYA                 55    0.144  26.458  -0.080   0.023   0.655   0.322
E P  4OFYA                 56    0.410  58.122  -0.624   0.004   0.420   0.576
E G  4OFYA                 57    0.502  39.507  -1.673   0.005   0.262   0.733
E N  4OFYA                 58    0.472  69.057  -1.642   0.004   0.085   0.910
E K  4OFYA                 59    0.497 102.253  -1.301   0.004   0.138   0.858
E R  4OFYA                 60    0.345  78.913  -0.599   0.004   0.616   0.381
B Y  4OFYA                 61    0.112  23.913  -0.585   0.002   0.816   0.182
E R  4OFYA                 62    0.256  58.510   0.042   0.001   0.900   0.099
B M  4OFYA                 63    0.109  21.831  -1.395   0.001   0.900   0.099
B V  4OFYA                 64    0.222  34.091  -0.891   0.001   0.900   0.099
B G  4OFYA                 65    0.298  23.453  -1.396   0.003   0.718   0.279
E S  4OFYA                 66    0.391  45.849  -1.246   0.004   0.420   0.576
E A  4OFYA                 67    0.507  55.849  -1.729   0.004   0.197   0.799
E A  4OFYA                 68    0.671  73.999  -1.777   0.004   0.085   0.910
E N  4OFYA                 69    0.580  84.941  -1.773   0.004   0.085   0.910
E G  4OFYA                 70    0.367  28.899  -1.089   0.005   0.262   0.733
E E  4OFYA                 71    0.416  72.640   0.149   0.004   0.616   0.381
B Y  4OFYA                 72    0.075  16.134   0.179   0.002   0.816   0.182
B N  4OFYA                 73    0.174  25.459   0.347   0.001   0.900   0.099
B L  4OFYA                 74    0.020   3.699   1.087   0.001   0.959   0.040
E E  4OFYA                 75    0.232  40.548   1.513   0.001   0.959   0.040
B I  4OFYA                 76    0.025   4.551   0.511   0.001   0.900   0.099
E S  4OFYA                 77    0.443  51.978   1.509   0.003   0.718   0.279
E N  4OFYA                 78    0.564  82.570   0.863   0.004   0.514   0.481
B V  4OFYA                 79    0.083  12.819  -0.023   0.005   0.262   0.733
E T  4OFYA                 80    0.491  68.060   1.080   0.005   0.336   0.660
E L  4OFYA                 81    0.542  99.313   0.223   0.005   0.262   0.733
E F  4OFYA                 82    0.648 130.074   0.400   0.004   0.138   0.858
B D  4OFYA                 83    0.187  26.875  -1.126   0.004   0.085   0.910
E D  4OFYA                 84    0.552  79.601  -0.145   0.004   0.138   0.858
B D  4OFYA                 85    0.160  23.070  -1.716   0.003   0.718   0.279
E D  4OFYA                 86    0.387  55.810   2.010   0.001   0.959   0.040
B F  4OFYA                 87    0.038   7.607   0.538   0.001   0.959   0.040
B A  4OFYA                 88    0.230  25.335   1.999   0.001   0.959   0.040
B C  4OFYA                 89    0.014   1.938   1.606   0.001   0.959   0.040
B Q  4OFYA                 90    0.183  32.737   1.674   0.001   0.959   0.040
B I  4OFYA                 91    0.030   5.457  -0.027   0.001   0.959   0.040
B S  4OFYA                 92    0.232  27.190   0.484   0.001   0.959   0.040
B E  4OFYA                 93    0.197  34.416   0.021   0.003   0.718   0.279
E S  4OFYA                 94    0.525  61.553  -0.499   0.004   0.138   0.858
E D  4OFYA                 95    0.662  95.337  -1.780   0.004   0.085   0.910
E H  4OFYA                 96    0.538  97.844  -1.810   0.004   0.085   0.910
E A  4OFYA                 97    0.512  56.422  -1.908   0.004   0.085   0.910
E K  4OFYA                 98    0.580 119.265  -1.058   0.004   0.138   0.858
E A  4OFYA                 99    0.385  42.383  -1.114   0.005   0.336   0.660
E V  4OFYA                100    0.359  55.148  -0.731   0.003   0.718   0.279
E V  4OFYA                101    0.275  42.298  -0.257   0.001   0.900   0.099
E S  4OFYA                102    0.367  43.036  -0.991   0.001   0.900   0.099
B S  4OFYA                103    0.239  28.058   0.199   0.001   0.900   0.099
E K  4OFYA                104    0.361  74.196   0.632   0.001   0.900   0.099
B A  4OFYA                105    0.028   3.075   0.187   0.001   0.959   0.040
E K  4OFYA                106    0.293  60.188   1.477   0.001   0.959   0.040
B L  4OFYA                107    0.021   3.808   1.094   0.001   0.959   0.040
B T  4OFYA                108    0.206  28.558   1.350   0.001   0.959   0.040
B V  4OFYA                109    0.030   4.580   0.433   0.001   0.959   0.040
E L  4OFYA                110    0.283  51.836   1.097   0.001   0.900   0.099
B V  4OFYA                111    0.144  22.148   0.641   0.004   0.616   0.381
E R  4OFYA                112    0.289  66.112   0.582   0.004   0.138   0.858
E P  4OFYA                113    0.482  68.467  -0.642   0.005   0.045   0.951
E T  4OFYA                114    0.652  90.460  -0.534   0.004   0.085   0.910
E P  4OFYA                115    0.418  59.357  -0.022   0.018   0.088   0.893
B P  4OFYA                116    0.152  21.540  -0.641   0.021   0.279   0.699
E K  4OFYA                117    0.539 110.811   0.260   0.003   0.718   0.279
B I  4OFYA                118    0.135  25.049  -0.036   0.002   0.816   0.182
E V  4OFYA                119    0.308  47.355  -0.780   0.021   0.756   0.223
E K  4OFYA                120    0.447  92.051  -1.111   0.004   0.420   0.576
E S  4OFYA                121    0.515  60.323  -0.656   0.004   0.085   0.910
E H  4OFYA                122    0.411  74.779  -1.695   0.005   0.045   0.951
E H  4OFYA                123    0.595 108.140  -0.784   0.004   0.085   0.910
E S  4OFYA                124    0.598  70.132  -0.717   0.004   0.420   0.576
B L  4OFYA                125    0.201  36.785   0.742   0.002   0.816   0.182
E K  4OFYA                126    0.454  93.326   0.701   0.001   0.900   0.099
B A  4OFYA                127    0.087   9.576   0.518   0.001   0.900   0.099
E I  4OFYA                128    0.451  83.491   1.045   0.002   0.816   0.182
E A  4OFYA                129    0.501  55.265   0.531   0.005   0.262   0.733
E G  4OFYA                130    0.700  55.074  -0.890   0.005   0.015   0.979
E D  4OFYA                131    0.465  67.079   0.843   0.005   0.045   0.951
E P  4OFYA                132    0.554  78.613   1.300   0.004   0.514   0.481
B I  4OFYA                133    0.050   9.268   0.557   0.001   0.900   0.099
E T  4OFYA                134    0.399  55.341   1.957   0.001   0.959   0.040
B Q  4OFYA                135    0.049   8.805   0.889   0.001   0.959   0.040
E S  4OFYA                136    0.337  39.461   1.643   0.001   0.959   0.040
B C  4OFYA                137    0.019   2.738   0.968   0.001   0.959   0.040
E L  4OFYA                138    0.375  68.644   1.693   0.001   0.959   0.040
B S  4OFYA                139    0.072   8.438  -0.805   0.001   0.959   0.040
E R  4OFYA                140    0.494 113.103  -0.190   0.004   0.616   0.381
E K  4OFYA                141    0.645 132.697  -1.512   0.004   0.138   0.858
E G  4OFYA                142    0.461  36.273  -1.723   0.005   0.045   0.951
E K  4OFYA                143    0.553 113.773  -0.381   0.005   0.045   0.951
B P  4OFYA                144    0.234  33.205  -0.715   0.004   0.085   0.910
E P  4OFYA                145    0.484  68.651   0.933   0.004   0.197   0.799
B P  4OFYA                146    0.074  10.515  -0.760   0.004   0.514   0.481
E T  4OFYA                147    0.367  50.917   1.262   0.001   0.900   0.099
B I  4OFYA                148    0.031   5.661   0.573   0.001   0.959   0.040
E G  4OFYA                149    0.261  20.533   1.889   0.001   0.959   0.040
B W  4OFYA                150    0.025   5.940   0.877   0.001   0.959   0.040
B A  4OFYA                151    0.204  22.437   1.289   0.001   0.959   0.040
B I  4OFYA                152    0.194  35.982   0.931   0.002   0.816   0.182
E A  4OFYA                153    0.594  65.481  -0.465   0.018   0.088   0.893
E S  4OFYA                154    0.682  79.895  -1.679   0.018   0.019   0.964
E D  4OFYA                155    0.650  93.636  -0.262   0.004   0.514   0.481
E E  4OFYA                156    0.565  98.671  -0.448   0.004   0.616   0.381
B H  4OFYA                157    0.201  36.507  -0.601   0.022   0.359   0.619
E G  4OFYA                158    0.441  34.667  -0.614   0.021   0.279   0.699
E K  4OFYA                159    0.510 104.907  -1.540   0.019   0.141   0.840
E H  4OFYA                160    0.533  96.916  -1.865   0.056   0.142   0.802
E I  4OFYA                161    0.424  78.495  -1.790   0.021   0.279   0.699
E V  4OFYA                162    0.432  66.322  -1.217   0.021   0.451   0.528
E S  4OFYA                163    0.357  41.887  -1.219   0.022   0.552   0.426
E W  4OFYA                164    0.361  86.821  -1.285   0.022   0.552   0.426
E L  4OFYA                165    0.344  62.968  -1.199   0.021   0.451   0.528
E G  4OFYA                166    0.394  31.024  -1.868   0.022   0.359   0.619
E E  4OFYA                167    0.501  87.595  -2.093   0.021   0.279   0.699
E S  4OFYA                168    0.342  40.082  -1.792   0.022   0.359   0.619
E R  4OFYA                169    0.380  86.906  -1.366   0.021   0.451   0.528
E S  4OFYA                170    0.314  36.848  -1.532   0.021   0.451   0.528
E K  4OFYA                171    0.387  79.606  -1.037   0.022   0.359   0.619
B F  4OFYA                172    0.303  60.712  -1.399   0.022   0.359   0.619
E G  4OFYA                173    0.361  28.395  -1.663   0.021   0.451   0.528
B G  4OFYA                174    0.302  23.744  -1.338   0.021   0.756   0.223
B I  4OFYA                175    0.207  38.276  -0.807   0.018   0.846   0.136
E H  4OFYA                176    0.360  65.502  -0.630   0.011   0.918   0.071
E A  4OFYA                177    0.347  38.184  -0.565   0.018   0.846   0.136
E K  4OFYA                178    0.362  74.546  -0.725   0.023   0.655   0.322
E P  4OFYA                179    0.383  54.390  -1.376   0.022   0.359   0.619
E E  4OFYA                180    0.551  96.242  -1.868   0.018   0.088   0.893
E I  4OFYA                181    0.564 104.377  -1.487   0.018   0.088   0.893
E S  4OFYA                182    0.481  56.326  -1.472   0.018   0.088   0.893
E Q  4OFYA                183    0.493  88.032  -1.520   0.020   0.205   0.775
E E  4OFYA                184    0.385  67.225  -0.611   0.004   0.420   0.576
B T  4OFYA                185    0.233  32.289  -0.709   0.003   0.718   0.279
B V  4OFYA                186    0.234  36.012  -0.558   0.002   0.816   0.182
B I  4OFYA                187    0.197  36.427  -0.467   0.002   0.816   0.182
B A  4OFYA                188    0.234  25.743  -1.548   0.003   0.718   0.279
E H  4OFYA                189    0.324  58.936  -1.028   0.022   0.552   0.426
B V  4OFYA                190    0.282  43.390  -1.008   0.022   0.359   0.619
E N  4OFYA                191    0.481  70.448  -1.264   0.020   0.205   0.775
E E  4OFYA                192    0.512  89.534  -1.233   0.021   0.279   0.699
E T  4OFYA                193    0.441  61.222  -1.083   0.022   0.359   0.619
E T  4OFYA                194    0.373  51.735  -1.004   0.021   0.451   0.528
E Q  4OFYA                195    0.467  83.317  -0.569   0.022   0.552   0.426
E V  4OFYA                196    0.387  59.559  -1.311   0.021   0.451   0.528
E E  4OFYA                197    0.465  81.288  -0.984   0.022   0.359   0.619
E E  4OFYA                198    0.394  68.797  -1.233   0.021   0.279   0.699
E G  4OFYA                199    0.500  39.326  -1.259   0.021   0.279   0.699
E G  4OFYA                200    0.312  24.523  -1.347   0.021   0.279   0.699
E N  4OFYA                201    0.472  69.057  -0.451   0.022   0.552   0.426
E N  4OFYA                202    0.327  47.917  -1.139   0.022   0.552   0.426
E S  4OFYA                203    0.294  34.398  -0.965   0.023   0.655   0.322
E R  4OFYA                204    0.326  74.585  -0.887   0.022   0.552   0.426
E E  4OFYA                205    0.490  85.620  -1.200   0.022   0.359   0.619
E D  4OFYA                206    0.522  75.220  -1.249   0.019   0.141   0.840
E S  4OFYA                207    0.536  62.843  -1.534   0.018   0.088   0.893
E S  4OFYA                208    0.448  52.494  -0.840   0.019   0.141   0.840
E I  4OFYA                209    0.504  93.314  -0.948   0.004   0.420   0.576
B Y  4OFYA                210    0.246  52.613  -1.190   0.004   0.514   0.481
E S  4OFYA                211    0.465  54.498  -1.033   0.004   0.616   0.381
B I  4OFYA                212    0.211  39.035  -0.986   0.003   0.718   0.279
E M  4OFYA                213    0.459  91.786  -0.683   0.003   0.718   0.279
B S  4OFYA                214    0.161  18.893  -1.478   0.004   0.616   0.381
E N  4OFYA                215    0.398  58.253   0.441   0.003   0.718   0.279
B L  4OFYA                216    0.068  12.451  -0.278   0.002   0.816   0.182
E S  4OFYA                217    0.368  43.118   0.609   0.001   0.900   0.099
B F  4OFYA                218    0.109  21.856  -0.441   0.001   0.900   0.099
E I  4OFYA                219    0.418  77.386   0.858   0.002   0.816   0.182
E P  4OFYA                220    0.499  70.879  -0.396   0.003   0.718   0.279
B R  4OFYA                221    0.162  37.098  -0.230   0.004   0.514   0.481
E P  4OFYA                222    0.509  72.284   0.136   0.005   0.336   0.660
E E  4OFYA                223    0.600 104.855  -0.185   0.004   0.197   0.799
E D  4OFYA                224    0.637  91.763  -1.333   0.005   0.045   0.951
E D  4OFYA                225    0.454  65.436  -1.047   0.005   0.045   0.951
E H  4OFYA                226    0.507  92.187  -0.222   0.005   0.336   0.660
B K  4OFYA                227    0.068  14.049  -1.095   0.002   0.816   0.182
E Y  4OFYA                228    0.390  83.322   1.897   0.001   0.959   0.040
B L  4OFYA                229    0.042   7.617   0.824   0.001   0.959   0.040
B I  4OFYA                230    0.246  45.417   1.624   0.001   0.959   0.040
B C  4OFYA                231    0.013   1.839   1.553   0.001   0.959   0.040
E I  4OFYA                232    0.236  43.660   1.832   0.001   0.959   0.040
B S  4OFYA                233    0.033   3.903  -0.536   0.001   0.959   0.040
E Q  4OFYA                234    0.406  72.422   0.566   0.001   0.900   0.099
B H  4OFYA                235    0.184  33.397  -0.975   0.004   0.514   0.481
E M  4OFYA                236    0.561 112.316  -0.557   0.004   0.138   0.858
E T  4OFYA                237    0.389  53.899  -2.031   0.004   0.085   0.910
E F  4OFYA                238    0.400  80.380  -0.696   0.004   0.138   0.858
E P  4OFYA                239    0.511  72.468  -2.059   0.004   0.085   0.910
E N  4OFYA                240    0.562  82.248  -1.196   0.018   0.047   0.935
E K  4OFYA                241    0.442  90.919  -1.436   0.004   0.138   0.858
E I  4OFYA                242    0.394  72.890  -0.452   0.004   0.514   0.481
E E  4OFYA                243    0.403  70.491  -0.589   0.003   0.718   0.279
E V  4OFYA                244    0.389  59.774  -0.686   0.002   0.816   0.182
B D  4OFYA                245    0.229  33.042   0.183   0.001   0.900   0.099
E S  4OFYA                246    0.366  42.872   0.650   0.001   0.900   0.099
B V  4OFYA                247    0.059   9.053   0.074   0.001   0.959   0.040
E K  4OFYA                248    0.330  67.902   1.739   0.001   0.959   0.040
B L  4OFYA                249    0.037   6.775   0.346   0.001   0.959   0.040
E S  4OFYA                250    0.314  36.848   1.257   0.001   0.959   0.040
B L  4OFYA                251    0.044   7.983   0.221   0.001   0.900   0.099
E R  4OFYA                252    0.337  77.173   1.105   0.002   0.816   0.182
B Y  4OFYA                253    0.261  55.669   0.290   0.004   0.420   0.576
E A  4OFYA                254    0.321  35.341   0.832   0.018   0.088   0.893
B P  4OFYA                255    0.201  28.522  -1.659   0.018   0.088   0.893
E Q  4OFYA                256    0.502  89.693   0.198   0.022   0.359   0.619
B H  4OFYA                257    0.281  51.096  -0.395   0.022   0.552   0.426
E H  4OFYA                258    0.515  93.624  -0.058   0.022   0.552   0.426
E H  4OFYA                259    0.522  94.897  -0.554   0.021   0.451   0.528
E H  4OFYA                260    0.512  93.042   0.702   0.021   0.279   0.699
E H  4OFYA                261    0.522  94.988  -0.369   0.018   0.088   0.893
E H  4OFYA                262    0.654 119.017  -2.252   0.003   0.003   0.994

Explain the output.  Go back.
```

---
